# Development of the “JPCC Barriers to Pediatric Colostomy Care Scoring System”: A Modified Delphi Study

**DOI:** 10.1002/wjs.70101

**Published:** 2025-09-23

**Authors:** Giulia Brisighelli, Catterina Bebington, Marion Arnold, Lindiwe Dyamara, Yentl Gamiet, Leila Hartford, Jane Hoole, Laura Obbes, Juan Scribante

**Affiliations:** ^1^ Department of Pediatric Surgery Chris Hani Baragwanath Academic Hospital School of Clinical Medicine Faculty of Health Sciences University of the Witwatersrand Johannesburg South Africa; ^2^ Johannesburg Pediatric Colorectal Clinic Chris Hani Baragwanath Academic Hospital Soweto South Africa; ^3^ Surgeons for Little Lives Johannesburg South Africa; ^4^ Division of Paediatric Surgery Red Cross War Memorial Children's Hospital Department of Surgery Faculty of Health Sciences University of Cape Town Cape Town South Africa; ^5^ Department of Paediatric Surgery Sefako Makgatho Health Sciences University Pretoria South Africa; ^6^ Wits Donald Gordon Medical Centre/Colorectal Unit Johannesburg South Africa; ^7^ Louise Obbes T/A Cura Care Nursing Services Windhoek Namibia

**Keywords:** assessment, challenges, children, colostomy, scoring system, stoma

## Abstract

**Background:**

In low‐ and middle‐income countries (LMICs), pediatric colostomy care is associated with significant clinical, social, and economic challenges that negatively impact patient outcomes. This study aimed to identify key barriers to pediatric colostomy care and to develop a scoring system for barriers to colostomy care.

**Methods:**

A modified Delphi study was conducted, involving caregivers of pediatric patients with colostomies and health care professionals managing such patients in Southern Africa. Forty individuals were invited to participate (20 caregivers and 20 health care professionals). In Round 1, participants were asked to list barriers to colostomy care via an online REDCap survey. Three authors (GB, CB, JS) thematically grouped identified barriers. In Round 2, participants rated the relevance of each statement: ≥ 75% agreement defined consensus. A virtual Round 3 refinement meeting was held with an expert panel to finalize the scoring system.

**Results:**

Of the 40 invited individuals, 23 (57.5%) participated in Round 1: 12 health care professionals (9 doctors, 2 nurses, and 1 with an unknown profession) and 11 caregivers. Sixteen participants completed Round 2, with consensus reached on all barrier statements. In Round 3, 4 nurses and 4 doctors reviewed and refined the statements, resulting in a final 36‐item JPCC barriers to colostomy care scoring system.

**Conclusion:**

This study presents the first scoring system specifically designed to measure barriers to pediatric colostomy care in Southern Africa. The scoring system offers a practical framework for research, clinical assessment, and advocacy. Further multicenter validation is recommended to assess its applicability across diverse settings.

## Graphical abstract

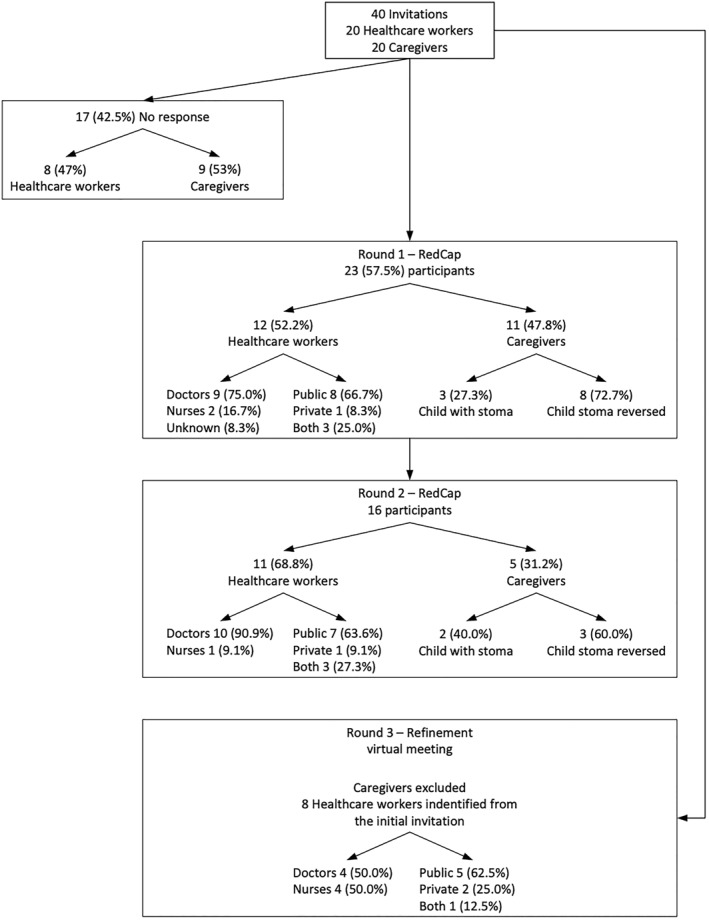

## 1 Introduction

In low‐ and middle‐income countries (LMICs), managing pediatric patients with colorectal conditions poses considerable challenges. Factors such as delayed presentation, suboptimal care, resource limitations, and discontinuity of follow‐up care contribute to poorer outcomes compared to high‐income countries (HICs) [1, 2, 3, 4].

In many cases, children with congenital colorectal conditions require a colostomy as an interim step before definitive surgical repair [5]. This is particularly true in LMICs, where sepsis and wound breakdown occur more frequently [5, 6, 7]. However, the barriers to colostomy care in children remain underexplored in both HICs and LMICs, with a limited understanding of the clinical, psychological, and practical challenges faced by caregivers and health care professionals [8, 9, 10, 11].

Caregivers, often faced with substantial barriers to colostomy care, may struggle with access to resources, technical skills, and psychological burdens [8, 10, 12]. Similarly, health care professionals in resource‐limited settings encounter systemic challenges in stoma management that complicate their ability to deliver optimal care [13]. Identifying and addressing these barriers is crucial to improving outcomes for pediatric patients with colostomies. Appropriate care and management can reduce complications such as peristomal skin damage and contribute to a smoother reversal process [11].

Despite the evident need for practical tools to support caregivers and health care professionals, no scoring system for quantifying barriers to colostomy care could be identified in the literature. There is a need for such a systematic diagnostic tool that is cognizant of resource‐limited settings to assist health care professionals and caregivers in delivering optimal colostomy care to patients. This study aimed to identify key barriers to caring for and managing a child from the perspectives of caregivers and health care professionals and to develop a scoring system for barriers to colostomy care in resource‐constrained settings in Southern Africa.

## 2 Methods

This study employed a modified Delphi method to identify key barriers to pediatric colostomy care [14, 15]. Ethical approval for this study was obtained from the Human Research Ethics Committee (Medical) (M230827) at the University of the Witwatersrand. Written informed consent was obtained from all participants.

Participants included caregivers of pediatric patients with colostomies attending the Johannesburg Pediatric Colorectal Clinic (JPCC) at Chris Hani Baragwanath Academic Hospital (CHBAH) and health care professionals managing such patients in South Africa, Namibia, and Zimbabwe. A purposive sampling method was employed to ensure the representation of relevant stakeholders. Although there are no universally accepted guidelines for the optimal number of participants in a modified Delphi study, Okoli and Pawlowski recommend a panel of 10–18 participants per stakeholder group [15]. To account for potential attrition and to ensure sufficient responses, 20 caregivers and 20 health care professionals were invited to participate. Caregivers were approached during follow‐up clinic visits, during which this study was explained and consent was obtained. Health care professionals were invited via email and provided consent electronically. Participants who agreed to participate were sent an anonymous link to a REDCap survey platform, along with up to four weekly email reminders to complete Rounds 1 and 2.

In Round 1, participants were asked to list at least six barriers they had experienced in the care and management of colostomies. Responses were collated and thematically grouped by three authors (GB, CB, and JS). When appropriate, similar barriers were merged into unified statements.

In Round 2, via an anonymous link to a REDCap survey platform, participants were presented with the identified barriers grouped in statements and asked to classify them as (a) relevant, (b) relevant but requiring editing, and (c) not relevant. If Option b were chosen, a free‐text window would appear, allowing the participant to reword the statement.

Consensus in Round 2 was defined as a rate of agreement of 75% or higher [16], calculated using the following formula:

$$\text{Rate}\;\text{of}\;\text{Agreement}=\frac{\left(\text{agreement}-\text{disagreement}\right)}{\left(\text{agreement}+\text{disagreement}+\text{indifferent}\right)}\times 100,$$

All barrier statements reached consensus in Round 2; thus, a formal third round was not required. However, to refine the scoring system further and to determine the appropriate response options, a virtual Round 3 was conducted. An expert panel consisting of eight health care professionals was identified from the initially invited group, and all accepted the invitation. During this session, all statements were reviewed, and the wording was edited to enhance clarity, readability, and generalizability.

## 3 Results

**FIGURE 1:**
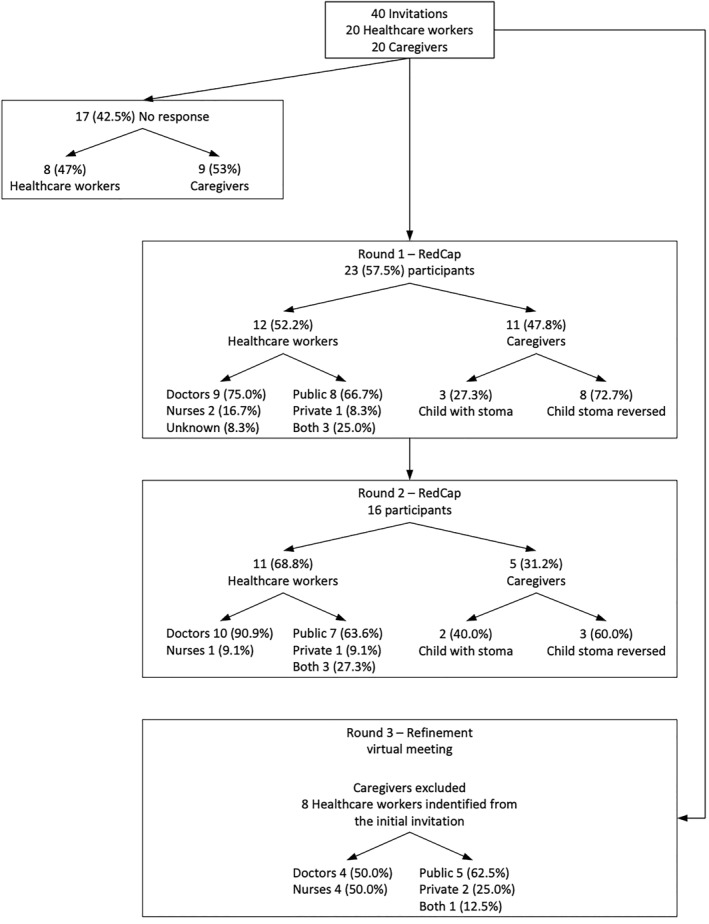
Sample realization.

Forty participants were invited to participate in this study; the sample realization for the three rounds is shown in Figure 1.

In Round 1, a total of 134 barriers were identified, with most of them being identified as barriers by more than one participant. The authors (GB, CB, and JS), individually and collectively, simplified and merged the responses into 35 statements. They were then organized into five main groups: stoma products, stoma care, social challenges, stoma care training, and financial (Table 1).

**TABLE 1:** Barriers identified in Round 1.

Stoma products
There are no stoma bags available at the clinic I am given enough stoma bags to last until my next visit The quality of the stoma bags is poor. For example, they leak or do not stick as they should I am given the wrong type of stoma bag for my child, (for example: I am given a urostomy bag [with a tap] instead of a colostomy bag) I am given the wrong size stoma bags for my child I am not given enough stoma care products, such as barrier seals or protective skin sprays, to last until the next clinic visit I know where to get stoma care products in the hospital
Stoma care
I am scared that I will hurt my child when caring for their stoma My family and friends are scared to help care for my child with a stoma I am worried my child's stoma bag will leak or show in public My child sometimes pulls their stoma bag off I find it difficult to know if there are problems with my child's stoma and how to handle the problems It is a lot of work to care for my child's stoma I battle to talk to or understand the doctors and nurses because of language difficulties My child's stoma makes their development delayed, for example, when they start crawling or walking I have limited access to water or electricity at times It is difficult to carry my child because of their stoma Is it difficult to see the stoma (part of the intestine) outside my child's skin There is a long waiting list at the hospital to close my child's stoma
Social challenges
My child is too shy to play with other children (reluctant) I am careful about my child playing with other children People discriminate against my child. For example, they cannot attend creche, school or play sport I am comfortable talking to anyone outside the clinic about having a child with a stoma
Stoma care training
I received adequate stoma care training I was made aware of the social challenges of having a child with a stoma The nurses working in the clinic know about stoma care If my child is admitted to the hospital for any reason, the ward nurses know about stoma care The doctors working in the clinic know about stoma care If my child is admitted to the hospital for any reason, the ward doctors know about stoma care I sometimes receive different or confusing stoma care advice from healthcare workers I have stoma care at my local hospital, and I don't need to travel to a big (academic) hospital
Financial
It is expensive to travel to the hospital or clinic for stoma care I have to spend extra money on stoma care products when I run out of product I am unable to work because of my child's stoma I sometimes miss clinic appointments because I do not have money to travel

In Round 2, 30 (86%) of the 35 statements were rated relevant. Table 2 summarizes the Round 2 rating of the statements.

**TABLE 2:** Round 2 rating of the statements. The statements that scored less than 75% for relevance are grey shaded.

Statement	Relevant	Relevant needs editing	Not relevant	Total
	*n* (%)	*n* (%)	*n* (%)	*n*
1. There are no stoma bags available at the clinic	11 (69%)	3 (19%)	2 (13%)	16
2. I am given enough stoma bags to last until my next visit	10 (62%)	4 (25%)	2 (13%)	16
3. The quality of the stoma bags is poor. For example, they leak or do not stick as they should	12 (75%)	3 (19%)	1 (6%)	16
4. I am given the wrong type of stoma bag for my child (for example: I am given a urostomy bag [with a tap] instead of a colostomy bag)	14 (87%)	1 (6%)	1 (6%)	16
5. I am given the wrong size stoma bags for my child	14 (87%)	2 (12.5%)	0 (0%)	16
6. I am not given enough stoma care products, such as barrier seals or protective skin sprays, to last until the next clinic visit	14 (87%)	2 (12.5%)	0 (0%)	16
7. I know where to get stoma care products in the hospital	16 (100%)	0 (0%)	0 (0%)	16
8. I am scared that I will hurt my child when caring for their stoma	13 (81%)	1 (6%)	2 (12.5%)	16
9. My family and friends are scared to help care for my child with a stoma	15 (94%)	1 (6%)	0 (0%)	16
10. I am worried my child's stoma bag will leak or show in public	15 (94%)	1 (6%)	0 (0%)	16
11. My child sometimes pulls their stoma bag off	16 (100%)	0 (0%)	0 (0%)	16
12. I find it difficult to know if there are problems with my child's stoma and how to handle the problems	14 (87%)	2 (13%)	0 (0%)	16
13. It is a lot of work to care for my child's stoma	14 (87%)	2 (13%)	0 (0%)	16
14. I battle to talk to or understand the doctors and nurses because of language difficulties	13 (86%)	0 (0%)	2 (13%)	15
15. My child's stoma makes their development delayed, for example, when they start crawling or walking	10 (62%)	2 (13%)	4 (25%)	16
16. I have limited access to water or electricity at times	13 (81%)	0 (0%)	3 (19%)	16
17. It is difficult to carry my child because of their stoma	13 (86%)	0 (0%)	2 (13%)	15
18. Is it difficult to see the stoma (part of the intestine) outside my child's skin	14 (93%)	0 (0%)	1 (7%)	15
19. There is a long waiting list at the hospital to close my child's stoma	12 (80%)	1 (7%)	2 (13%)	15
20. My child is too shy to play with other children (reluctant)	11 (73%)	2 (13%)	2 (13%)	15
21. I am careful about my child playing with other children	13 (86%)	1 (7%)	1 (7%)	15
22. People discriminate against my child. For example, they cannot attend creche, school or play sport	14 (93%)	0 (0%)	1 (7%)	15
23. I am comfortable talking to anyone outside the clinic about having a child with a stoma	14 (93%)	1 (7%)	0 (0%)	15
24. I received adequate stoma care training	11 (73%)	3 (20%)	1 (7%)	15
25. I was made aware of the social challenges of having a child with a stoma	13 (86%)	1 (7%)	1 (7%)	15
26. The nurses working in the clinic know about stoma care	12 (80%)	2 (13%)	1 (7%)	15
27. If my child is admitted to the hospital for any reason, the ward nurses know about stoma care	13 (86%)	2 (13%)	0 (0%)	15
28. The doctors working in the clinic know about stoma care	15 (100%)	0 (0%)	0 (0%)	15
29. If my child is admitted to the hospital for any reason, the ward doctors know about stoma care	13 (86%)	2 (13%)	0 (0%)	15
30. I sometimes receive different or confusing stoma care advice from healthcare workers	14 (93%)	1 (7%)	0 (0%)	15
31. I have stoma care at my local hospital, and I don't need to travel to a big (academic) hospital	13 (86%)	0 (0%)	2 (13%)	15
32. It is expensive to travel to the hospital or clinic for stoma care	14 (93%)	1 (7%)	0 (0%)	15
33. I have to spend extra money on stoma care products when I run out of product	11 (73%)	3 (20%)	1 (7%)	15
34. I am unable to work because of my child's stoma	12 (80%)	2 (13%)	1 (7%)	15
35. I sometimes miss clinic appointments because I do not have money to travel	14 (93%)	0 (0%)	1 (7%)	15

Table 3 summarizes the edits suggested in Round 2. Of the 11 suggestions for editing, 9 were general comments, whereas only 2 were specific requests for editing. The adjusted relevant ratings of statements 1, 3, 15, 24, and 33 are 14 (87%), 14 (87%), 12 (75%), 12 (80%), and 14 (93%), respectively. Therefore, all 35 statements were rated as relevant.

**TABLE 3:** Suggested edits suggested by participants.

	Participants		Suggestions
Statement	Caregivers *n* (%)	Health care professionals *n* (%)	
1	2 (66%)	1 (33%)	“Most of the bags don't last for a long time, also different stoma bags for different age groups and season will be better …. This also includes the number of bags given to kids should also go with age too. Barriers and other supplies besides the bags it's self should be given at all visits by all dispensers and not just that one particular nurse. And some advice and tricks on how to keep the bag longer especially if you're far from the clinic/Bara hospital.” “There are limited stoma bags at the clinic and we can only fetch them from the hospital, it would be easier if they were also available at our local clinics. That could help us save money.” “There is often no stoma bags at the clinic.”
2	2 (50%)	2 (50%)	“I mostly receive enough stoma bags till next visit.” “Size is important in keeping the bag longer and also how the stoma is situated on a child.” “We are given 10 stoma bags to last for a month and that is not enough especially with an active child the stoma bag can last for 2 days max.” “I am often not given enough stoma bags to last until the next visit.”
15	1 (100%)	—	“My child's stoma delays their development.”
24	1 (33%)	2 (66%)	“Maybe specify ‘before being discharged or later at the clinic’.” “Define exactly what is meant by stoma care training.” “I had some sort of training while still in NICU but later saw that things are done differently at the stoma clinic.”
33	—	3 (100%)	“Stoma care products are not readily available over the counter at pharmacies: a script is required. Think this needs editing.” “Stoma products not readily available so if parents run out, skin excoriation from double nappy use.” “When I run out of stoma products I have to spend extra money that I cannot always afford to spend.”

During Round 3, the panel reviewed each statement for clarity, neutrality, and relevance. Particular attention was given to removing overly emotive or subjective language (such as feelings, fears, or personal perceptions), with the goal of maximizing generalizability and avoiding response bias. Statements that reflected emotional reactions, such as worry, fear, or embarrassment, were either reworded or moved into a renamed category—social challenges, fears, and perceptions—where such themes could be addressed more appropriately. One statement was divided into two separate items, bringing the total to 36 statements.

The panel also reached consensus on using a 3‐point Likert scale for scoring, with the following response options:

- 1 = Not a challenge
- 2 = A moderate challenge
- 3 = A significant challenge

**FIGURE 2:**
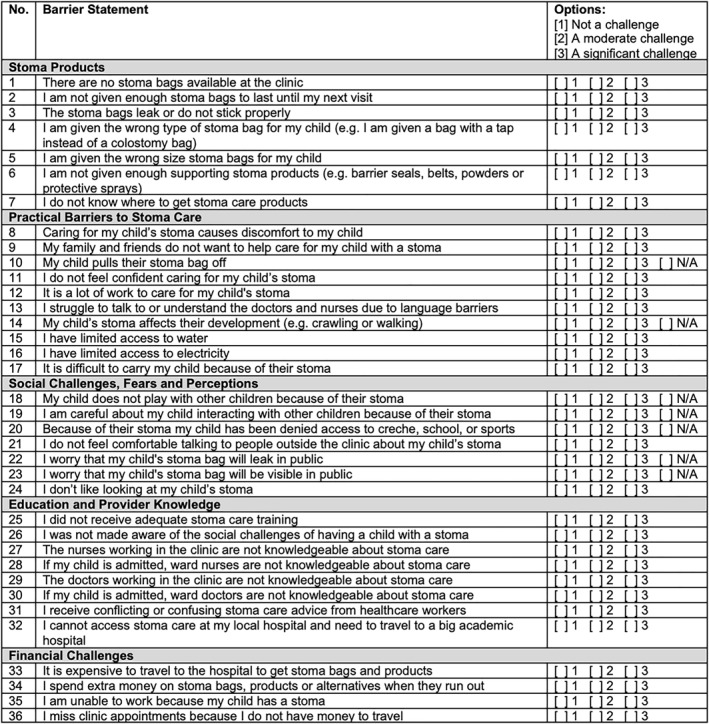
JPCC barriers to colostomy care scoring system.

Lastly, a “not applicable” option was added to the Likert scale in statements where not all caregivers may experience the barrier. The final scoring system, the “JPCC barriers to colostomy care scoring system,” is presented in Figure 2.

## 4 Discussion

The JPCC barriers to colostomy care scoring system, developed through consensus and based on the experience of caregivers and health professionals, provides a pragmatic approach to measuring challenges in limited‐resource settings in Southern Africa. Its standardization enables easy comparison across hospitals, supports implementation research, and helps flag households in need of targeted support. Although it has not yet undergone formal validation, its development marks a noteworthy first step that could assist health care professionals in LMICs. It may also serve as an advocacy mechanism, offering real‐world data to support training, policy reform, and funding requests. Importantly, the scoring system incorporates the perspectives of both caregivers, who often face isolation, stigma, a lack of stoma training, and resource scarcity [8, 10, 11], and health care professionals, who must navigate systemic constraints [4].

Ensuring consistent access to the correct type, size, and quantity of stoma products has a direct impact on patient outcomes and quality of life [8, 11]. The barriers regarding stoma products identified in our study have also been described in a Ugandan study [8].

Issues such as water and electricity shortages require systemic solutions beyond the control of individual health care professionals. However, other practical barriers to stoma care, such as caregiver confidence and language challenges, once quantified, can be more immediately addressed through improved counseling, tailored education, and greater awareness of these specific needs within clinical settings.

The social challenges, fears, and perceptions identified in our study are not limited to LMICs. A 2024 national study from the United Kingdom highlighted the psychological and social burden that stomas impose on children and their families [11]. Although caregivers felt reasonably well informed about the surgical and practical aspects of care, they reported a deficit in information related to the psychological and lifestyle implications of stoma formation [11]. Notably, psychosocial issues, including bag leaks, stigma, and lifestyle restrictions, were rated as more distressing than surgical complications [11]. Identifying and addressing the social challenges, fears, and perceptions of caregivers will ensure that patients receive holistic stoma care.

Identifying shortcomings in caregiver education and training of health care professionals related to stoma care for those directly and indirectly involved in stoma care allows for facilitating focused stoma care education and training. For example, strengthening capacity at the primary care level could help reduce the burden on tertiary and quaternary centers, decrease travel costs for families, and improve attendance at follow‐up appointments. It will allow for outside‐the‐box solutions, as illustrated by Mazira et al. [8], where they integrated structured peer support into stoma care education to complement formal health care provider training in Uganda [8].

Financial challenges not only affect the family's economic stability but also contribute to missed clinic appointments and delayed care [4]. Addressing these barriers requires integrated, family‐centered stoma care strategies that include improving access to products, decentralizing basic stoma care where possible, and considering financial support mechanisms for vulnerable families [8].

Taken together, these findings provide important insights into the multifaceted barriers faced by families and health care providers in delivering optimal colostomy care in Southern Africa. Future iterations of the scoring system could explore incorporating differential weighting of individual barriers to reflect their relative impact on care, which could be used as a tool to prioritize stoma‐related surgery in resource‐constrained settings, as well as expand its application and validation across different settings.

Translation of the scoring system into local languages may improve its accessibility at the point of care. This would enable caregivers from diverse linguistic backgrounds to fully engage with the tool.

This study has several limitations. For practical reasons, caregivers were recruited from an urban central hospital, which may limit insight into the experiences of caregivers in rural areas, where barriers are likely to be greater. Although this study aimed to reflect the Southern African context, most participants were from South Africa, which may limit the regional generalizability. A larger, multicenter study across different countries and health care levels is needed to validate the scoring system.

## 5 Conclusions

This study presents the first scoring system designed to capture barriers to pediatric colostomy care in a limited‐resource context. By integrating the perspectives of both caregivers and health care professionals, the scoring system provides a much‐needed framework to document, quantify, and ultimately address the systemic, financial, educational, and psychosocial challenges these caregivers and families face. Its development marks an important step toward standardizing care, improving outcomes, and informing policy in resource‐limited settings. Broader implementation of the scoring system in Southern Africa and other LMICs has the potential to drive targeted interventions, foster collaboration, and ensure that children living with stomas receive the comprehensive, dignified care they deserve.

## Author Contributions

**Giulia Brisighelli:** conceptualization, methodology, investigation, formal analysis, project administration, writing – original draft, writing – review and editing. **Catterina Bebington:** conceptualization, methodology, investigation, writing – review and editing. **Marion Arnold:** writing – review and editing, supervision. **Lindiwe Dyamara:** investigation, data curation. **Yentl Gamiet:** writing – review and editing. **Leila Hartford:** writing – review and editing. **Jane Hoole:** investigation, writing – review and editing. **Laura Obbes:** investigation, resources. **Juan Scribante:** conceptualization, methodology, supervision, writing – review and editing.

## Ethics Statement

All authors comply with the journal's ethical policies.

## Consent

Informed consent was obtained from all individual participants included in this study.

## Conflicts of Interest

The authors declare no conflicts of interest.

## Data Availability

The data that support the findings of this study are available from the corresponding author upon reasonable request.
